# Idiopathic Slow Transit Constipation: Pathophysiology, Diagnosis, and Management

**DOI:** 10.3390/medicina60010108

**Published:** 2024-01-06

**Authors:** Luke J. Vlismas, William Wu, Vincent Ho

**Affiliations:** 1 Deptartment of Gastroenterology, Campbelltown Hospital, Campbelltown, NSW 2560, Australia; williamwenhao.wu@health.nsw.gov.au (W.W.); vincent.ho@health.nsw.gov.au (V.H.); 2 School of Medicine and Public Health, University of Newcastle, Newcastle, NSW 2308, Australia; 3 School of Medicine, Western Sydney University, Campbelltown, NSW 2560, Australia

**Keywords:** slow transit constipation, constipation, colon, dysmotility, enteric nervous system, manometry, pathophysiology, diagnosis, management, prokinetcs

## Abstract

Slow transit constipation (STC) has an estimated prevalence of 2–4% of the general population, and although it is the least prevalent of the chronic constipation phenotypes, it more commonly causes refractory symptoms and is associated with significant psychosocial stress, poor quality of life, and high healthcare costs. This review provides an overview of the pathophysiology, diagnosis, and management options in STC. STC occurs due to colonic dysmotility and is thought to be a neuromuscular disorder of the colon. Several pathophysiologic features have been observed in STC, including reduced contractions on manometry, delayed emptying on transit studies, reduced numbers of interstitial cells of Cajal on histology, and reduced amounts of excitatory neurotransmitters within myenteric plexuses. The underlying aetiology is uncertain, but autoimmune and hormonal mechanisms have been hypothesised. Diagnosing STC may be challenging, and there is substantial overlap with the other clinical constipation phenotypes. Prior to making a diagnosis of STC, other primary constipation phenotypes and secondary causes of constipation need to be ruled out. An assessment of colonic transit time is required for the diagnosis and can be performed by a number of different methods. There are several different management options for constipation, including lifestyle, dietary, pharmacologic, interventional, and surgical. The effectiveness of the available therapies in STC differs from that of the other constipation phenotypes, and prokinetics often make up the mainstay for those who fail standard laxatives. There are few available management options for patients with medically refractory STC, but patients may respond well to surgical intervention. STC is a common condition associated with a significant burden of disease. It can present a clinical challenge, but a structured approach to the diagnosis and management can be of great value to the clinician. There are many therapeutic options available, with some having more benefits than others.

## 1. Introduction

Constipation is the symptom of unsatisfactory defecation and can occur either in association with identifiable triggers or as a primary chronic condition. Chronic idiopathic constipation (CIC) is a common condition affecting a significant proportion of adults worldwide. A 2011 meta-analysis by Suares and Ford found a worldwide prevalence of 14%, with variations geographically [1]. CIC is one of the most common gastrointestinal complaints and reasons for an ambulatory review, and frequently impacts on quality of life [2,3,4,5]. Several known risk factors for CIC exist, most notably a female gender and an increased age, particularly age over 65. A higher prevalence of low socioeconomic status has also been observed [1,4,6].

Disease phenotypes of CIC include impaired evacuation due to dyssynergic defecation (DD), colonic dysmotility resulting in slow transit constipation (STC), or constipation without evidence of abnormal defecation or delayed colonic transit (normal transit constipation; NTC) [3,4,7,8]. Normal transit constipation is the most common phenotype and frequently overlaps with constipation-predominant irritable bowel syndrome (IBS-C) [7].

STC is the least common of the CIC phenotypes, however, variations in prevalence occur depending on the setting, and the true population prevalence is difficult to determine as the majority of patients with NTC are successfully managed in the primary care setting and many patients with chronic constipation do not require advanced investigation to define STC [4,7]. The prevalence of patients with STC within a population of patients with CIC has been reported to range between 15–30% [9], giving an estimated STC prevalence of 2–4% in the general population, based on the abovementioned worldwide prevalence of CIC.

STC is sometimes classified as a functional gut disorder, as in the Rome Foundation’s classification of disorders of gut-brain interaction [10]; however, there is objective evidence of the disease in these patients based on motility studies and pathologic examination of colectomy specimens [2,4,7,11,12], and the condition is likely to be a neuromuscular disease of the colon [7]. Although the aetiology of STC remains unclear, our understanding is evolving, and hormonal and autoimmune mechanisms have been proposed [7,13,14]. The microbiome may also play a role in the aetiology of some patients, but its overall contribution to the pathophysiology remains unclear [15].

STC may be challenging to manage and, at its extreme, may require surgical intervention. It frequently results in poor quality of life and significant psychosocial stress, and also commonly results in high health care burden with frequent presentations to health care [6]. It can be difficult to distinguish between the phenotypes of constipation clinically, and although management strategies for each are similar initially, the management of those who fail standard first-line therapies differs greatly. Therefore, having an algorithmic approach to the diagnosis and management is of vital importance to the clinician.

This review article summarises the findings of a literature review on the topic of slow transit constipation in adults, providing readers with an overview of the pathophysiology, diagnostic modalities, and management options for STC. It also proposes a framework for approaching the diagnosis and management.

## 2. Definitions and Classification

Constipation is generally defined as unsatisfactory defecation, characterised by increased stool firmness, reduced frequency of bowel movements, and/or difficult evacuation [3,7,8,16,17]. The term chronic generally refers to abnormalities that are present for three months or longer, and the development of STC is generally insidious, other than in certain secondary causes such as spinal cord injury. The aetiologies of constipation are numerous but can be classified as either primary or secondary. Primary constipation, often synonymous with idiopathic constipation, relates to intrinsic colonic or anorectal dysfunction, whereas secondary constipation occurs as a result of structural abnormalities, systemic disease, or medications [7,17].

The American Gastroenterological Association (AGA) classifies chronic constipation into three phenotypes: DD; STC; and NTC [3]. DD results in an impaired rectal evacuation and may or may not have a secondary delayed colonic transit due to rectal outlet obstruction. NTC is constipation without evidence of DD and with a normal colonic transit time. Some patients with CIC, particularly those with NTC, have an overlap with IBS-C, which is predominantly characterised by abdominal pain in addition to bowel disturbances [3,18].

STC occurs due to colonic dysmotility, resulting in delayed colonic transit times not due to DD. A proportion of these patients have a co-existing upper gastrointestinal dysmotility, with one study reporting a delayed gastric emptying in 34%, a delayed small bowel transit in 10%, and both in 8% [8]. The term colonic inertia refers to a state of severely impaired colonic motility with an absence of post-prandial increased motor activity or a lack of response to stimulant laxatives [2,7,17,18].

The conditions defined by AGA’s classification appear in the Rome IV criteria as functional constipation and functional defecation disorders, with IBS again being defined separately but may co-exist [10]. However, the Rome IV Criteria are based on symptoms alone and are not as useful when discussing STC as there is no requirement for colonic transit studies for the diagnosis in this classification system, and the majority of patients with functional constipation have normal transit times [4,10].

## 3. Pathophysiology

### 3.1. Normal Physiology of the Colon

The primary function of the colon is water reabsorption and waste transportation towards the rectum where it is excreted as stool via the anus [7,12]. These functions rely on complex interactions between the endocrine, nervous, and muscular systems.

#### 3.1.1. Control of Colonic Function

The majority of lower gastrointestinal function is under involuntary control; however, the process of defecation has voluntary and involuntary mechanisms. The colonic function is maintained primarily by neural and hormonal input [7].

The motor function is coordinated by input from the enteric nervous system, which contains both sympathetic and parasympathetic nerves. The enteric nervous system interfaces with the colonic smooth muscle via the colonic myenteric plexuses and the interstitial cells of Cajal (ICC) [2,7,12]. The ICCs act as the pace-making cells of the colon, mediating the signals of the enteric nervous system and the colonic smooth muscle, and are essential in the generation and propagation of electrical slow waves [4]. Both stimulating (e.g., serotonin [5-hydroxytryptamine, 5-HT], and acetylcholine) and inhibitory (e.g., nitric oxide) neurotransmitters are released by the enteric nerves to produce peristaltic waves [7].

The endocrine system contributes to both the motility and the fluid/electrolyte function of the colon. Hormones such as cholecystokinin and motilin contribute to the post-prandial increase in colonic motor activity (the gastrocolic reflex), and hormones, such as the thyroid hormone, interact with the enteric nervous system to regulate intestinal motility. Similar to its action in the kidneys, aldosterone also helps to regulate sodium and water reabsorption in the colon [4,12].

#### 3.1.2. Fluid and Electrolyte Homeostasis

The colon contributes to fluid and electrolyte homeostasis, reabsorbing 1–2 L of fluid per day [4,7]. The amount of water reabsorbed is a time-dependent process, and hence, the states that result in a delayed evacuation of faecal material result in harder, smaller stools [7].

Sodium is actively reabsorbed through multiple active transport channels. Countering this, chloride, and subsequently sodium, are secreted through chloride channels, though this function is largely inactive in the normal state, resulting in a net reabsorption of fluid and electrolytes [7]. Water is passively reabsorbed or secreted in response to osmotic gradients created by these processes in balance with the osmotic pressure of the intestinal contents [7].

#### 3.1.3. Motor Function

The normal colonic transit times in adults range from 20–72 h [7]. Multiple different types of motor patterns occur in the colon and anorectum and can be propagating or non-propagating [4,12]. Non-propagating motor patterns serve as segmentation and mixing functions and aid in fluid and electrolyte reabsorption [4,7,12,18]. Non-propagating motor patterns are low-amplitude and occur as random contractions, as well as short-length peristaltic contractions, both in the antegrade and retrograde directions. These short peristaltic contractions are the result of the spontaneous myogenic slow waves created by ICCs. Retrograde peristaltic contractions act as a normal physiologic brake: in the right colon, it delays ileocaecal emptying and increases nutrient absorption in the small bowel; and in the left colon, it increases colonic transit time and subsequently water reabsorption, as well as assisting with the control of continence [12].

Propagating motor patterns result in powerful contractions which propel contents from the right to left colon towards the anus, resulting in mass movements, which are the main type of propulsive motility of the colon [4,7,12,18]. High-amplitude propagating contractions (HAPCs) can be seen with high-resolution manometry and are the manometric description of mass movements [4,12]. HAPCs occur spontaneously a few times each day, typically in the morning, and can be triggered or augmented by certain triggers, such as eating (the gastrocolic reflex) [4,7]. Pan-colonic pressurisations are simultaneous pressure increases across the length of the colon, which occur in unison with internal anal sphincter relaxation, resulting in the urge to defecate and facilitating the evacuation of bowel motions [12].

Defecation is the process of the evacuation of stool from the rectum via the anus. The process begins with rectal filling, followed by the coordination of relaxation of the muscles of the pelvic floor and anal sphincter and contraction of the abdominal wall and rectum [7].

### 3.2. Pathophysiology of Constipation

The disruptions of physiologic mechanisms leading to constipation vary greatly between the different phenotypes. The pathophysiology of NTC is unclear but is likely multifactorial. DD results from an impaired coordination of the muscles of defecation, leading to an impaired relaxation or paradoxical contraction of the anus, and/or an inadequate rectal and abdominal propulsive force. In some patients, DD may result in delayed colon transit time due to rectal outlet obstruction [4,7].

STC is thought to be a neuromuscular disorder of the colon, and dysmotility can be demonstrated by various means. Manometric studies have displayed a reduction in the number or complete absence of HAPCs, an impaired or absent gastrocolic reflex, and an overall reduced motor activity of both propagating and non-propagating patterns [4,7,12]. Ambulatory 24 h colonic manometry has demonstrated a similar nocturnal colonic pressure activity in STC compared with the controls but with an attenuation or absence of the normal increase in motor activity on waking [15]. An increase in retrograde peristaltic contractions has also been demonstrated during manometry, resulting in an exaggerated colonic break function [19]. Transit studies have shown delayed emptying, particularly of the proximal colon, and some patients may also have co-existing dysmotility of the stomach or small bowel [4,8].

Although the aetiology of STC remains unclear, several pathophysiologic features have been observed in these patients, and therefore, our understanding is evolving [7,13]. There is a strong female predominance and hormonal contributions to the aetiology have been hypothesised. Colectomy specimens have demonstrated increased progesterone receptors, which correlate with alterations to the contractile and inhibitory G-proteins [13]. Autoantibodies have been demonstrated in the pathology specimens of a small proportion of patients with gastrointestinal dysmotility, including STC, suggesting a possible autoimmune aetiology in some patients [14].

Patients may also have abnormal or reduced numbers of interstitial cells of Cajal [4,7,15,18], and in the majority of cases of patients who have undergone colectomy for refractory STC, histological examination shows an abnormal or reduced number of ICCs [11]. Additionally, reduced amounts of excitatory neurotransmitters within myenteric plexus neurons have been demonstrated [2,7].

Differences in the gut microbiome and metabolites have been observed in patients with STC, including an increased prevalence of methanogenic flora [15,20]. Although the overall contribution to the pathophysiology of STC and aetiological mechanisms remain unclear, methane gas, a product of the fermentation of dietary fibre by intestinal bacteria, has been shown to delay gastrointestinal transit and impair motility in animal models [15]. An examination of the microbiome may also act as a potential biomarker in the diagnosis of STC [20].

## 4. Diagnosis

Making a confident diagnosis of STC can be challenging, as symptoms overlap substantially with other phenotypes of CIC and secondary causes of constipation. Therefore, having an algorithmic approach to the diagnosis and management can be of great use to the clinician.

### 4.1. Differential Diagnoses

Prior to making a diagnosis of STC, it is important to consider and exclude the differential diagnoses of chronic constipation.

#### 4.1.1. Other Phenotypes of Primary Chronic Constipation

As mentioned above, the phenotypes of CIC can be classified as one of DD, STC, or NTC [3]. A fourth phenotype is sometimes described, where patients have overlapped DD and STC [8], though these patients would be classified as having DD with delayed colonic transit in the AGA’s classification system. For most of these patients, their delay in transit is due to rectal outlet obstruction and can be overcome with management of the DD; however, some patients may have true colonic dysmotility.

IBS-C is also a differential diagnosis to consider, and if a patient describes abdominal pain as their predominant symptom, then this may be the more appropriate diagnosis, particularly if there is no evidence of DD or delayed colonic transit in the investigations. The phenotypes of CIC can co-exist with a diagnosis of IBS-C, most commonly NTC [18]. Table 1 lists the phenotypes of primary chronic constipation.

#### 4.1.2. Secondary Causes of Chronic Constipation

Prior to making a diagnosis of idiopathic STC, secondary causes should be considered, and the reversible risk factors addressed. The secondary causes of constipation may cause constipation either by inducing colonic dysmotility or by other pathophysiologic mechanisms.

Because colonic motility occurs through an interaction between hormonal, neuronal, and muscular systems, most of the secondary causes of STC are metabolic or neuromuscular disorders, as well as from medications that interact with these systems. Neurologic disorders are common secondary causes of STC and may be conditions that affect the central nervous system, such as Parkinsons disease, multiple sclerosis, or stroke, the peripheral nervous system, as in diabetic enteric neuropathy, or a combination of the two, as can occur in spinal cord injury. Additionally, some neurologic conditions may cause an overlap with STC and DD; this commonly occurs from spinal cord injury [2,3,4,7,18,21]. Table 2 lists the secondary causes of STC.

Other than processes that result in colonic dysmotility, other secondary causes of constipation should also be considered in the initial assessment. For example, a mechanical obstruction, such as from malignancy, stricture, or rectocoele, can obstruct the passage of faeces and cause constipation [3,7,18]. Other conditions associated with constipation, including psychiatric disorders, such as depression and eating disorders, cognitive impairment, immobility, cardiac disease, and non-coeliac gluten sensitivity, should also be considered [3,18,22].

### 4.2. Clinical Assessment

The purpose of the initial assessment is to exclude secondary causes, elicit any red flags, and characterise the nature and severity of the patient’s constipation to allow for a correct classification of their constipation phenotype [3,4,6]. A review of a patient’s medical history and medication list is an effective way to screen for secondary causes of constipation and may be addressed to improve symptoms without the need for advanced investigations. Red flags should be screened for, and if present, should prompt an investigation with a colonoscopy and/or cross-sectional imaging, primarily to exclude colorectal cancer [3,4].

A characterisation to determine the timing of onset, associated features, frequency of bowel motions, and description of stool form can be of use. The Bristol Stool Form Scale (BSFS) values < 3 correlate with delayed colonic transit time, whereas the frequency of defecation may not correlate well with the colonic transit time, particularly if not in the extreme [3,4,23,24]. In addition to the core features of hard, infrequent, and/or difficult-to-pass stools, patients with CIC may have a range of symptoms, including a sensation of an anorectal blockade, a feeling of incomplete evacuation, painful defecation, a need for digitation, abdominal pain, bloating, nausea, and vomiting [4,8,17]. Patients with STC typically experience a reduced urge to defecate and may have associated abdominal pain, nausea, and vomiting; however, it is difficult to distinguish STC from the other phenotypes of CIC by history alone [6,7]. Additionally, because some patients have extra-colonic gastrointestinal dysmotility, some associated symptoms relate more to upper gastrointestinal conditions, such as gastroparesis [8]. Because STC frequently causes psychosocial stress and impacts quality of life, it is important to assess the impact that the disease is having on a patient [6].

A clinical assessment can be useful to exclude DD. Some features of the history are more suggestive of this phenotype, including a sensation of an anorectal blockade, a feeling of incomplete evacuation, or a need for digitation [3]. The rectal examination findings of increased anal tone, impaired anal sphincter relaxation or paradoxical contraction, and/or decreased perineal descent have been shown to be an effective diagnostic tool for DD, with a sensitivity and specificity of 75% and 87%, respectively [3,18,25].

### 4.3. Investigations and Diagnostic Workup

Further investigation may not be required after initial assessment if there are no red flags present and the patient responds to first line management.

#### 4.3.1. Investigations to Rule out Secondary Causes

A colonoscopy is frequently performed in patients for the investigation of constipation, though in the absence of red flag features, a colonoscopy is often of low yield and may not be required [4]. However, a colonoscopy should be performed to exclude colorectal cancer if the patient has any red flags, is required as part of a bowel cancer screening program, or is refractory to medical management and is being considered for surgery [3]. Similarly, an investigation with cross-sectional imaging, such as an abdominal CT, may be appropriate if structural causes, such as intra-abdominal malignancy, are suspected from the initial assessment. Laboratory investigations can add value in a subset of patients, including screening for hypercalcaemia and hypothyroidism, though these are uncommon causes in those whose primary complaints are constipation [6].

#### 4.3.2. Investigations for Primary Constipation

Further investigation for suspected idiopathic constipation is generally only required for those who have failed simple laxative therapy. In this situation, an evaluation for DD or STC is important as these phenotypes are more commonly difficult to manage [17]. If patients with suspected CIC have not responded adequately to simple laxatives, a localisation to either the colon or the anorectum allows for the initiation of appropriate management [7].

If available, an assessment of anorectal function should be performed prior to colon transit studies to identify if DD is present, particularly if suspicion is high based on the clinical assessment. High-resolution anorectal manometry is the gold standard for diagnosing DD [4]. Other available tests of anorectal function include rectal balloon expulsion test, anal electromyography, and defecography [3,4,8,18].

##### Assessment of Colonic Motility

After excluding a rectal outlet obstruction and reversible secondary causes of constipation, an assessment of colonic transit is the next step in the workup of suspected STC and is essential to make the diagnosis [6,18]. In order to perform the testing of colonic motility, medications that alter transit times should be ceased prior in order to assess the true intrinsic colonic motility [3]. There are a number of methods of assessing colonic motility available in clinical practice, as well as those which are generally only performed in a research setting.

The radio-opaque marker test is often the standard diagnostic test used, which is widely available and simple to perform [3,4,7]. In this test, a capsule containing 20 radio-opaque markers is swallowed, and a plain film abdominal radiograph is taken 5 days later, with retention of 5 or more markers indicating slow transit. Some capsules contain a different number of markers, so a cut-off of 20% of the original number of markers is generally used. Although this test performs well in identifying STC, the number of retained markers does not correlate well with the severity or quality of life [4]. The Metcalf method is an alternative method for performing the radio-opaque marker test, which is able to approximate the total and segmental colonic transit times, which involves taking capsules on consecutive days. The number of retained markers on an X-ray the day after, both totally and segmentally, are counted [18].

Colonic scintigraphy is an alternative method that provides the total and segmental colonic transit times but is frequently less available than the radio-opaque marker test in clinical practice [3,4,7]. For this test, the patients consume a radio-isotope-labelled meal, and the transit time is calculated by making timed measurements of the residual radioactivity [4]. The scintigraphic images at 24 and 48 h are able to define the delayed colonic transit, and the results are given as a percentage of the radioactivity remaining in each colonic segment. By 48 h, a separation between patients with and without STC can be demonstrated, with the upper limit of normal being defined as the mean +/− 2 SD in the healthy controls [26]. Whole gut scintigraphy can also be used to assess for co-existing extra-colonic dysmotility.

In clinical practice, wireless motility capsules are the third most commonly used but are generally only available in limited settings, such as research centres. These capsules measure the chemical properties of the intestinal contents along the gastrointestinal tract to determine the transit time. They are able to provide information on transit through the stomach and small bowel as well but are unable to provide information on segmental colonic transit [3,4,7].

These three techniques used to measure colonic transit times are comparable in accuracy and have correlated well when performed on patients with constipation [3]. High-resolution colonic manometry is generally only performed in a research setting for clinical trials and studies on physiology but provides additional detail about the motor function of the colon [27].

**Figure 1:**
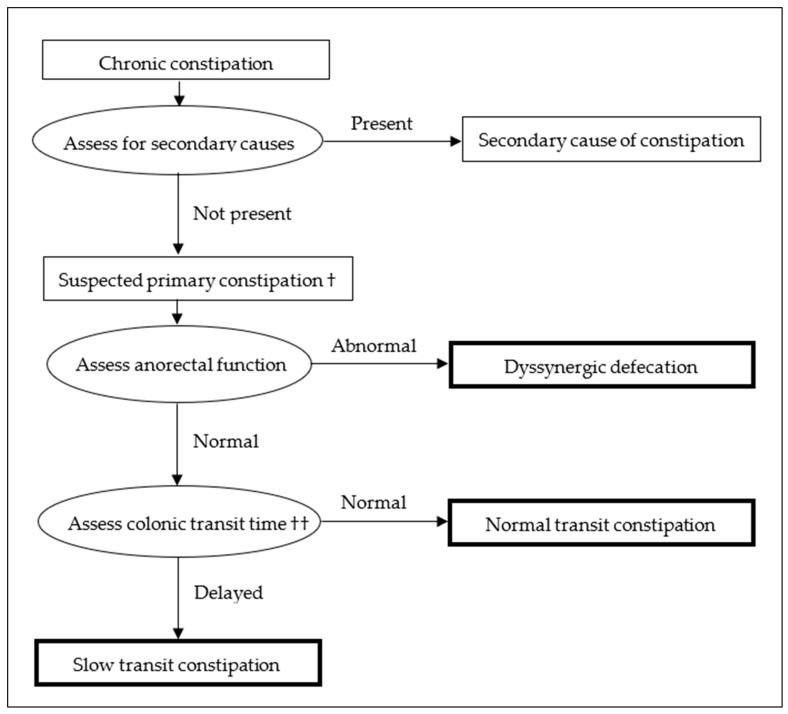
Diagnostic algorithm for slow transit constipation. † Simple laxatives should be trialled, and further investigations only performed in those who do not respond. †† If anorectal function testing is not available, it may be reasonable to proceed with colonic transit studies if suspicion of DD is not high based on clinical assessment, but testing should be pursued if there is persisting difficulty with management.

Figure 1 provides an approach to the diagnosis of STC and the other constipation phenotypes.

## 5. Management

There are several different management options for constipation, including dietary, pharmacologic, interventional, and surgical. A large proportion of people with constipation are managed by simple measures, such as fibre supplementation and standard laxatives, and these treatments should precede the use of the advanced investigations of anorectal and colonic functions listed above.

There is much overlap between the constipation phenotypes in the treatments available, with the exception of DD, which is best managed non-pharmacologically. If DD is identified by the testing of anorectal function, anorectal biofeedback, and pelvic floor physiotherapy are the most effective treatment, and these patients are commonly refractory to pharmacologic therapy [4,7].

One of the challenges in managing patients with STC is a lack of evidence specific to those with confirmed delayed colonic transit times, particularly for the pharmacologic trials. The inclusion criteria for most of the trials do not require an assessment of colonic transit; instead, patients are defined as having either functional constipation or CIC, and thus the trials would include patients with different constipation phenotypes. Given that patients with STC are more likely to be refractory to therapy compared to those with NTC, it is likely that the trials for more advanced therapies, such as prokinetics, do include a significant proportion of patients with STC, and the trials that have performed an assessment of transit time reflect this. However, it may be unclear how effective the therapies are in those with confirmed STC.

Each of the different management options, including their effectiveness in STC, will be discussed.

### 5.1. Lifestyle, Dietary and Fibre Supplementation

Increasing oral hydration is often recommended, but in the absence of dehydration, this has not been beneficial [3,4]; however, many fibre supplements and laxatives are recommended alongside increased oral hydration. Exercise has been shown to improve gastrointestinal symptoms and the quality of life in patients with IBS [3,4], but studies have shown mixed results regarding the effects of exercise on colonic transit time, and there is limited data on its effect in those with STC. However, increasing physical activity, and particularly addressing inactivity, may increase gut transit times [4,28,29,30,31].

Fibre supplementation may alter the water content and consistency of the stools, as well as affect the gastrointestinal microbiota by their prebiotic effect. Although soluble fibre supplements can be an effective treatment for many patients with CIC [3,4,32], they may have a limited benefit in slow transit constipation and may worsen the patient’s symptoms, such as bloating and abdominal pain [18]. This lack of efficacy is demonstrated by delayed transit times in the colon transit studies which define STC, a method that requires the consumption of a high volume of fibre to perform [33].

Probiotics can be recommended; however, the role of probiotics in the management of CIC is unclear [4]. The proposed mechanisms of benefit in constipation include the restoration of non-pathogenic gastrointestinal microbiota and the increased bacterial production of lactate and short-chain fatty acids. For STC, their effectiveness is similarly unclear, though a 2014 meta-analysis by Dimidi et al., investigating the effects of probiotics in patients with functional constipation, showed a significantly improved whole gut transit time, stool frequency, and stool consistency; however, there was significant heterogeneity between the studies and the high risk of bias, and the outcomes in patients with STC were not observed [34].

### 5.2. Pharmacologic

There are multiple pharmacologic targets for the treatment of constipation, including gut motility, secretory function of the colon, and faecal fluid composition [4,7].

#### 5.2.1. Osmotic Laxatives

Osmotic laxatives passively draw water into the intestinal lumen by osmotic gradients, which increases stool water content and facilitates colon propulsion [3,7,18]. Polyethylene glycol (PEG) containing osmotic laxatives is commonly used as first-line pharmacotherapy for CIC [4,35]. Lactulose, a non-absorbable carbohydrate, is also commonly used; however, PEG was shown to be more effective than lactulose for CIC in a 2010 meta-analysis by Lee-Robichaud et al., and its use may be limited by its common side effects of bloating and flatulence [35,36]. Magnesium oxide is an alternative that has been shown to improve the frequency of bowel movements and quality of life when compared with the placebo, but patients should be monitored for hypermagnesaemia, particularly those with renal impairment [18,35,37]. The goal of osmotic laxative therapy is to produce soft but not liquid stools, with doses being titrated to achieve this [3]. Patients with STC may or may not respond to osmotic laxatives, but these should be trialled in all patients with CIC, preferably prior to undertaking advanced investigations.

#### 5.2.2. Stimulant Laxatives

Stimulant laxatives are irritant substances that directly stimulate the afferent nerves or the gastrointestinal smooth muscle to induce gut motility, including colonic HAPCs [7]. Several stimulant laxatives, including bisocodyl, sodium picosulfate, and senna, were shown to improve constipation and quality of life in CIC [4,35,36,37,38,39]. The side effects commonly experienced include abdominal pain and cramping, and diarrhoea [35]. Similarly, stimulant suppositories, such as bisacodyl and glycerin, can be used to improve stool consistency and the ease of defecation in chronic constipation [3].

The long-term safety of stimulant laxatives is commonly questioned in clinical practice. However, there is no evidence that long-term use has any negative impact on colonic motility or that it induces physiologic dependence [3,4,33,40,41].

Although effective in other forms of constipation, this class may have limited effectiveness in those with STC, as studies have demonstrated a reduced colonic motor response to these agents, and colonic inertia is defined by a lack of response to these agents [33]. However, a trial of stimulant laxatives, typically in combination with other classes such as osmotic laxatives, should be attempted.

#### 5.2.3. Stool Softeners

Stool softeners are surfactants that reduce the surface tension of faecal material and promote water retention within the stool. The common agents in this class are docusate and liquid paraffin. They may provide some benefit to patients with constipation but often provide little improvement to patients with CIC when used in isolation and are shown to be inferior to psyllium in improving stool frequency. Their effectiveness in treating STC is unclear [3,18].

#### 5.2.4. Secretagogues

Secretagogues target the chloride channels and induce electrolyte and fluid secretion, thereby increasing the faecal water content [4,7,35]. The increase in fluid content both accelerates colonic transit and improves the ease of defecation [3]. Lubiprostone, linaclotide, and plecanatide are the available agents in this class and can be effective in CIC, though their availability varies between regions, and their use may be limited by the side effects, particularly diarrhoea [16,35]. A 2023 meta-analysis by Chang et al., comparing lubiprostone to a placebo in patients with CIC, demonstrated an increased number of spontaneous bowel movements by 2/week. However, there was no subgroup analysis performed on the patients with STC. Although the increased intestinal fluid content induced by secretagogues may accelerate gastrointestinal transit times [35], their effectiveness in the management of STC have not been studied in depth.

#### 5.2.5. Bile Acid Transporter Inhibitors

Elobixibat is a new treatment currently under development. It is an inhibitor of ileal bile acid transport, which induces a state of bile acid malabsorption, increasing colonic fluid secretion and promoting colonic motility [4]. It has shown promise in patients with CIC, and a 2019 post-hoc analysis of two phase-three trials by Nakajima et al. showed efficacy in patients with severe constipation and implied a benefit in those with STC. Using the criteria of <2 bowel movements per week and BSFS <3, which can be independent predictors for STC, suggested it is effective in those with both STC and NTC [42,43]. However, further studies are required to better define its effectiveness in those with STC.

#### 5.2.6. Prokinetics

Prokinetics stimulate gastrointestinal motility, inducing intestinal peristalsis and augmenting propagating contractions [7], and are generally the most effective medical therapies for patients with STC. There are several different classes of prokinetic agents in use.

5-HT4 receptor agonists facilitate acetylcholine release from enteric neurons and include multiple agents in the class, such as prucalopride, cisapride, tegaserod, mosapride, and itopride [3,4,7,16,35]. Prucalopride has a potent colonic prokinetic effect and has the strongest evidence to support its use in STC. It is also the most commonly used of the 5-HT4 agonists for CIC in clinical practice. Unlike cisapride, tegaserod, and itopride, prucalopride has not shown any relevant electrocardiographic changes, nor has it been associated with adverse cardiovascular effects [16,18,44,45]. A 2011 meta-analysis by Ford and Suares reviewed the efficacy of prucalopride in CIC, analysing seven RCTs that compared prucalopride with a placebo in 2639 participants with CIC. This meta-analysis showed a clinical response of 28.3% vs. 13.3% in those treated with prucalopride vs. a placebo, respectively, corresponding to a NNT of six [46]. One 2002 RCT by Emmanuel et al., which was included in the above meta-analysis, performed whole-gut transit studies using the radio-opaque marker test on all the participants before and after the treatment period and also performed sub-group analyses on those with STC vs. NTC. Of the total 74 participants, a majority (58%) were classified as STC. Prucalopride at a dose of 1 mg daily reduced the number of retained markers in all the patients when compared with a placebo. A significant reduction in the number of retained markers in those with STC, but not those with NTC, was also demonstrated. 22% of the prucalopride-treated patients with delayed transit at the baseline improved to normal transit times, compared with only 5% in the placebo group [47].

Other 5-HT4 receptor agonists have also been used in STC. Cisapride has both cholinergic and serotonergic effects. It has a pan-gastrointestinal prokinetic effect, with more of an effect on upper gastrointestinal motility than colonic, and may have a greater role in patients with co-existing gastroparesis [48]. Mosapride has shown effectiveness in patients with secondary causes of STC, such as parkinsonism and diabetes [49,50]. Tegaserod was previously used for the management of constipation but has been removed from the market and is no longer available [51]. Velusetrag and naronapride are also 5-HT4 receptor agonists which are currently undergoing clinical trials [4].

Colchicine is an anti-inflammatory medication commonly used for the treatment of gout, which has a dose-dependent side effect of inducing diarrhoea and can be used in the management of CIC. The exact mechanism by which it results in diarrhoea is unclear, but it ultimately induces intestinal secretions and colonic motility [33,52]. A 2010 RCT by Taghavi et al. compared colchicine 1 mg daily to a placebo in patients with confirmed STC and showed significantly improved symptom scores and increased frequency of spontaneous bowel movements in the treatment group, with 26/30 participants treated with colchicine having an acceptable symptomatic response [52].

Misoprostol is a synthetic prostaglandin-E_1_ analogue used to treat and prevent non-steroidal anti-inflammatory drug-related peptic ulcers that has the common side effect of diarrhoea. In addition to its effect on gastric acid production, it also increases gastrointestinal fluid production and motility [33,53]. A 1997 open-label trial by Roarty et al. observed the effect of oral misoprostol at a starting dose of 200 µg TDS in 18 patients with refractory constipation. An intolerance to the medication due to abdominal discomfort was common; 6/18 patients dropped out prior to the completion of the study period, but 10/12 participants who tolerated the medication had an improved frequency of bowel movements [53].

The motilin receptor agonist erythromycin has prokinetic properties, which are more pronounced in the upper gastrointestinal tract, but can also stimulate distal colonic motility in a patient with reduced plasma motilin [6]. However, a 1998 trial by Bassotti et al., investigating the effect of intravenous erythromycin on colonic motility in 18 participants with STC, concluded that it had little prokinetic effects in the colon, although some increased activity in the distal colon was demonstrated at a low dose [54]. Anecdotally, some experts have seen the benefit of erythromycin in STC, and a trial may be reasonable in patients, particularly those with co-existing upper gastrointestinal involvement [6].

Cholinesterase inhibitors stimulate upper and lower gastrointestinal motility, with parenteral neostigmine commonly used in the treatment of acute intestinal pseudo-obstructions and oral pyridostigmine having shown a benefit in the management of chronic and recurrent intestinal pseudo-obstruction [55,56,57]. Pyridostigmine reduces colonic transit times and improves the symptoms in patients with chronic constipation and those with secondary causes of slow transit constipation [58,59,60,61,62]. However, studies on those with idiopathic STC are lacking. A 2010 study by O’Dea et al. investigated the efficacy of pyridostigmine in patients with severe constipation or recurrent pseudo-obstruction, which included six patients with STC. This study showed a benefit in only one patient, with the remaining five ceasing the medication, four of which ultimately required colectomy for refractory STC [57]. Its use may be limited because of the cholinergic side effects, but serious adverse events are rare [57,60]. Although larger randomised trials are required to better assess its effectiveness in patients with idiopathic STC, based on its efficacy in similar conditions, physiologic plausibility, and safety profile, it may be reasonable to trial pyridostigmine for patients with idiopathic STC who have failed other prokinetic medications.

Some prokinetic agents that have an effect on the upper gut, like metoclopramide and domperidone, have no effect on colonic motility and are not useful in STC.

Table 3 summarises the above advanced pharmacological therapies used in STC, including the recommended doses and regimens.

### 5.3. Interventional and Surgical

There are a number of interventional and surgical methods that have been used for the treatment of medically refractory STC, and the choice depends on a patient’s profile, disease phenotype, and severity.

#### 5.3.1. Faecal Microbiota Transplant

Faecal microbiota transplantation (FMT) involves the delivery of donor faecal matter to the recipient’s gastrointestinal lumen and has been beneficial for a number of different gastrointestinal conditions. FMT can be delivered by a number of different techniques, including a nasointestinal tube, a colonoscopy, or an enema. A 2017 RCT by Tian et al. investigated the use of FMT in patients with STC who showed significantly improved symptoms with FMT compared with conventional treatment, with a clinical cure rate of 36.7% and 13.3% [63], respectively. Unfortunately, the 2018 long-term follow-up study of this cohort showed a loss of efficacy over time in some patients [64].

#### 5.3.2. Electrical Stimulation

Sacral nerve stimulation has been used for the treatment of STC to induce colonic propagating contractions. Earlier uncontrolled studies suggested a benefit in the patients with STC; however, subsequent higher-quality studies have shown that it was not associated with improved symptoms of constipation or an increase in colonic transit times, as well as high rates of patient dissatisfaction and risks of complications, such as infection, and haematoma [65,66,67,68].

Transcutaneous electrical stimulation has been used to improve the symptoms associated with STC, with more experience in the paediatric population than in adults. A 2016 Cochrane Review on its use in children with STC was unable to draw any conclusions due to the low quality of evidence and the high risk of bias in the included studies [69]. There are a few studies in the adult population; however, a 2017 RCT by Yang et al. compared transcutaneous electrical stimulation to sham intervention in 28 women with STC which showed a significant improvement in the symptoms and defecation frequency [70]. Overall, the effectiveness of transcutaneous electrical stimulation in the treatment of STC remains unclear, but it may be beneficial to some patients and is a safe therapy with no serious adverse effects.

Colonic pacing with intramuscular electrode placement is an experimental treatment for STC, which has shown some promise in animal models and a limited number of humans, but more research is required before its use can be recommended [71].

#### 5.3.3. Acupuncture

Acupuncture can be a safe treatment option in the management of CIC and can improve symptoms of constipation, however there is a high degree of heterogeneity in the studies investigating its use [72]. There is limited evidence in its use in patients with STC; however, a 2013 RCT by Peng et al. showed significant improvement in stool frequency with deep puncture acupuncture therapy when compared with shallow puncture and western medication groups at the six month follow up visit, but the outcomes at the earlier assessments were not significantly different [73].

#### 5.3.4. Transanal Irrigation

Transanal irrigation can be beneficial to patients with CIC, as well as those with secondary constipation, including from a spinal cord injury. A 2015 meta-analysis by Emmett et al. investigated the effectiveness of transanal irrigation in patients with functional constipation and demonstrated a 50.4% response rate across the seven uncontrolled studies (254 participants), although substantial heterogeneity was present [74]. Despite not being well-investigated in patients with idiopathic STC, it is a safe and well-tolerated adjunct and may be reasonable to trial in agreeable patients.

#### 5.3.5. Antegrade Colonic Enemas

The creation of cecostomy or appendicostomy, either through percutaneous endoscopic cecostomy or appendiceal conduits, respectively, allows for the use of antegrade colonic enemas to promote colonic emptying [3,17,18]. The choice between these two modalities depends on the patient’s profile and the surgeon’s preference, but appendiceal conduits are generally preferred in the paediatric population [33]. These interventions are less invasive than colectomy, particularly endoscopic cecostomy, which can be performed under local anaesthetic and conscious sedation and can improve symptoms of constipation in the majority of patients [18]. There is a larger pool of evidence in the paediatric population than in adults, but two uncontrolled cohort studies in adults have demonstrated a benefit. A 2004 retrospective study by Lees et al. reported on 32 patients with refractory constipation caused by STC, DD, or mixed STC/DD who underwent cecostomy/appendicostomy conduit creation, with satisfactory function achieved in 47% [75]. A 2001 prospective study by Rongen et al. observed 12 patients with medically refractory STC who underwent cecostomy/appendicostomy conduit creation and showed an improved median defecation frequency from 1/week to 1/day; there were no major complications, but 4/12 ultimately required colectomy due to persisting constipation [76]. Although there is limited evidence available in adults, the results of the above studies suggest a benefit in a population of patients who may otherwise require colectomy, and the creation of cecostomy/appendicostomy does not appear to affect their suitability for further surgeries.

A number of different irrigation solutions are used for antegrade colonic enemas, including tap water, saline, PEG, glycerin, and mineral oil [77].

#### 5.3.6. Surgery

Various forms of surgery have been used for medically refractory STC, but the most common and most effective is a total colectomy, either with ileorectal anastomosis or ileostomy formation [4,7,78]. Ileostomy without colectomy can be considered in patients who are at high operative risk [2]. Segmental colectomy has been used for treatment but may be ineffective if the remaining colon is also disordered and does not perform better than ileorectal anastomosis in trials, and so total colectomy is generally the preferred surgery [2,18].

Surgical intervention is rarely indicated in patients with constipation because the optimal patient selection is of vital importance, but in the correct circumstances, outcomes can be good, and the patients’ symptoms may respond well [4,7]. Prior to the consideration of surgery, reversible secondary causes need to be excluded, and the patients should have medically refractory STC and have exhausted pharmacologic options. A 2017 systematic review by Knowles et al. found an 86% average satisfaction rate, with rates from individual studies ranging from 81–89% [78]. However, the adverse event rate is not insignificant, with a total complication rate of 24%, comprising a mortality of 0.4%, a re-operation rate of 13%, and a small bowel obstruction rate of 15%. Additionally, patients commonly have long-term symptoms following surgery, including abdominal pain in 30–50%, bloating in 10–40%, recurrence of constipation in 10–30%, and diarrhoea in 5–15% [2,78].

Colectomy is only suitable for patients with proven STC and is not suitable for those with NTC [3,4]. Surgery is rarely indicated in patients whose phenotype is DD unless their symptoms are refractory to biofeedback and pelvic floor physiotherapy. When DD and STC co-exist, DD should be treated prior to the consideration of surgery. If surgery is to be considered despite addressing DD, an ileostomy is preferred over an ileorectal anastomosis [4,33].

For patients with both STC and extra-colonic gastrointestinal dysmotility, it can be hypothesised that a patient’s upper gastrointestinal dysmotility may improve with colectomy for the management of STC; however, a 2001 cohort study by Mollen et al., investigating the effects of colectomy on gastric emptying in patients with STC, showed no difference before and after surgery on the gastric emptying time [79]. Therefore, the use of colectomy should undergo careful consideration in those with both colonic and extra-colonic dysmotility, as these patients have lower satisfaction rates [18]. Similarly, patients with isolated STC, whose predominant symptoms are abdominal pain or bloating, are more likely to have persisting symptoms. In both of these circumstances, a trial with a loop ileostomy may be performed to determine the suitability to proceed with colectomy [18].

**Figure 2:**
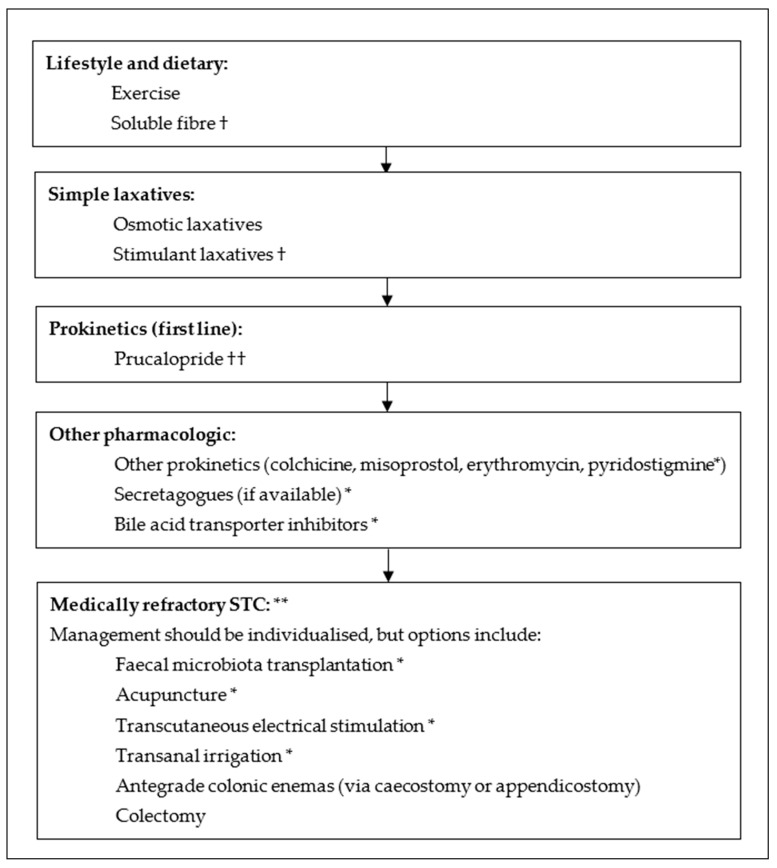
Management algorithm for slow transit constipation. † If there is no improvement with these therapies, then they should not be emphasised and discontinuation should be considered due to minimal benefit in STC. †† If there is no response to prucalopride, the other 5-HT4 agonists may be trialled, but may be similarly ineffective. The use of prokinetic agents of other classes may have more benefit in this circumstance. * Limited evidence in STC to guide management but may be beneficial in some patients. ** Assure that DD has been excluded and STC confirmed.

Figure 2 provides an algorithm for the management of patients with STC, and Table 4 summarises the therapeutic trials which have reported on patients with confirmed STC.

### 5.4. Future Directions

Future studies should continue to investigate the pathophysiology and therapeutic options for patients affected by STC. Continued advancements in our understanding of the underlying pathophysiology leading to STC will help to guide future studies, with the ultimate aim of identifying therapeutic targets. These areas include the neuromuscular function of the colon, as well as the microbiome.

Given the relative paucity of evidence for pharmacotherapy in patients with confirmed STC, it would be beneficial if future trials could be performed to address this, with inclusion/exclusion criteria designed to exclude the other constipation phenotypes.

Pharmacologic agents currently under investigation include the 5-HT4 receptor agonists velusetrag and naropride, which are currently undergoing clinical trials for use in CIC [4]. It would be beneficial if the available secretagogues, including lubiprostone, linaclotide, and plecanatide, underwent further studies into their effectiveness in the management of patients with STC, given their benefit in patients with severe CIC. Similarly, the bile acid transporter class shows promise with elobixibat, but further research is required to evaluate its efficacy in patients with STC.

Although sacral nerve stimulation appears to provide no benefit, transcutaneous electrical stimulation may hold some promise in patients with STC but requires further evaluation with larger randomized controlled trials. Additionally, although colonic pacing with intramuscular electrodes is currently experimental, its use may prove to be a useful therapy to avoid surgery in otherwise refractory cases but requires further evaluation.

## 6. Conclusions

STC is a significant condition that has an estimated prevalence of 2–4% in the general population. It frequently impacts quality of life and is associated with significant psychosocial stress and high healthcare costs. Our understanding of the pathophysiology is evolving, but it is likely to be a neuromuscular disorder of the colon. Observed abnormalities include reduced motor activity on manometry; delayed emptying on transit studies; hormonal changes, abnormal neurotransmitter activity, and reduced ICCs on histology; and alterations of the microbiome. The underlying aetiology is uncertain, but autoimmune and hormonal mechanisms have been hypothesised. It can be a challenging condition to manage, but a structured approach to the diagnosis and management can be of great value to the clinician. Therapeutic options include lifestyle and dietary changes, laxatives, pharmacotherapy, and interventional therapies, with prokinetic agents generally providing the most effective medical therapy for these patients. Though it is rarely required, medically refractory STC may respond well to colectomy.

## Figures and Tables

**Table 1:** Phenotypes of primary chronic constipation.

Chronic Idiopathic Constipation
Dyssynergic defecation (with or without delayed colonic transit)
Slow transit constipation
Normal transit constipation ^†^
Constipation predominant irritable bowel syndrome ^†^

^†^ IBS-C and NTC often co-exist.

**Table 2:** Secondary causes of slow transit constipation.

Neurologic Disorders
Parkinson’s disease
Multiple sclerosis
Stroke
Spinal cord injury
Diabetic enteric neuropathy
Myopathies
Systemic sclerosis
Amyloidosis
Metabolic disorders
Hypothyroidism
Hypercalcaemia
Uraemia
Diabetes mellitus
Medications
Opiates
Anticholinergics (e.g., antidepressants, antispasmodics, antipsychotics)
Dopaminergics (e.g., levodopa, dopamine agonists, antipsychotics)
Calcium channel blockers
5-HT3 antagonists

**Table 3:** Summary of advanced pharmacotherapies used in slow transit constipation.

Medication	Mechanism	Recommended Regimen	Comments
Prokinetics			
Prucalopride	5-HT4 agonist	1–2 mg daily, oral Maximum 4 mg/day	Typical first line prokinetic in STC.
Cisapride	Cholinergic; 5-HT4 agonist	10 mg QID, oral	May be preferred in patients with co-existing gastroparesis.
Mosapride	5-HT4 agonist	5 mg TDS, oral	Evidence for use in secondary causes of STC but limited in idiopathic STC.
Colchicine	Uncertain	1 mg daily, oral	Limited evidence in STC, but available evidence suggests benefit.
Misoprostol	Prostaglandin analogue	200 µg TDS, oral Maximum 2400 µg/day	May be limited by abdominal discomfort. Limited evidence in STC.
Erythromycin	Motilin receptor agonist	40 mg TDS, oral or IV Maximum 2 g/day	Conflicting data for benefit in STC.
Pyridostigmine	Cholinesterase inhibitor	60 mg TDS, oral Maximum 720 mg/day	Physiologically plausible and beneficial in similar conditions (pseudo-obstruction and secondary STC), but limited evidence in idiopathic STC.
Bile acid transporter inhibitors			
Elobixibat	Bile acid transporter antagonist	5–15 mg daily, oral	Limited evidence in STC, but available evidence suggests benefit.
Secretagogues			
Lubiorostone	Chloride channel agonist	24 µg BD, oral	Limited evidence in STC, but effective in severe CIC.
Linaclotide	CFTR agonist	72–145 µg daily, oral Maximum 290 µg/day	Limited evidence in STC, but effective in severe CIC.
Plecanatide	CFTR agonist	3 mg daily, oral	Limited evidence in STC, but effective in severe CIC.

**Table 4:** Summary of therapeutic trials reporting on slow transit constipation.

Author, Year, Article Type	Treatment	Population	Study Characteristics	Outcomes
Emmanuel et al. [47] 2002 RCT	Prucalopride 1 mg	Females aged over 18 with functional constipation. Whole gut transit was performed on all participants, and subgroup analysis on those with STC was performed.	74 (all female) participants, 43 classified with STC. Overall, 37 treatment, 37 placebo. Of those with STC, 22 treatment, 21 placebo.	Prucalopride reduced the number of retained markers in all patients when compared with placebo by 11.2 vs. 1.1 (*p* < 0.05), respectively. Prucalopride significantly reduced the number of retained markers in those with STC by 17.3 (*p* < 0.05), but the change in baseline by 1.6 in NTC was not significant.
Taghavi et al. [52] 2010 RCT	Colchicine 1 mg daily	Patients with chronic constipation who had STC confirmed with colon transit time.	60 participants (47 female). 30 treatment, 30 placebo.	Colchicine significantly improved symptom scores and increased frequency of spontaneous bowel movements. 26/30 participants treated with colchicine had an acceptable symptomatic response.
Roarty et al. [53] 1997 Open-label trial	Misoprostol 200 µg TDS. Dose titration based on response and tolerance was allowed, with a range of 400–2400µg/day.	Adults with chronic constipation refractory to available medical therapy, who had STC confirmed with colonic transit time.	18 participants (15 females). All received treatment.	Intolerance to misoprostol due to abdominal discomfort was common, with 6/18 patients dropping out prior to completion of the study period. 10/12 participants who tolerated misoprostol had improved frequency of bowel movements. Of the patients who tolerated the medication, mean bowel movement frequency improved from 11.25 to 4.8 days (*p* = 0.0004).
Bassotti et al. [54] 1998 Open-label	Erythromycin 50, 200, and 500 mg IV	Females with severe constipation with confirmed STC with colonoscopically positioned manometric probe, and effects of treatment on motility were assessed.	18 participants (all female). All received placebo infusion followed by treatment.	Erythromycin had little prokinetic effects in the colon, although some increased activity in the distal colon was demonstrated at a low dose.
Bharucha et al. [60] 2013 RCT	Pyridostigmine 60 mg TDS initially. Increased every three days to a maximum of 120 mg TDS, based on effect and tolerance.	Diabetic patients with CIC. All patients had scintigraphy to determine colonic transit time, and 13/30 participants had confirmed slow transit.	30 patients (22 female) 16 received treatment, and 14 received placebo. Of the 13 participants with STC, eight received pyridostigmine and five placebo.	Significantly increased colonic transit overall (*p* < 0.01), as well as improved stool frequency and consistency (*p* = 0.04). 7/8 vs. 2/5 patients with STC had normalisation of colonic transit times with pyridostigmine vs. placebo, respectively.
O’Dea et al. [57] 2010 Open-label	Pyridostigmine 10 mg BD initially, increased if required.	Adults with refractory STC or recurrent pseudo-obstruction who were being considered for colectomy.	13 overall, six with STC. All patients received treatment.	Of those with STC, 1/6 participants had improved symptoms. 4/5 who had no benefit ultimately underwent colectomy.
Tian et al. [63] 2017 RCT	FMT 100 mL by nasointestinal tube daily for six days, in addition to conventional therapy. Compared unblinded to conventional therapy alone.	Adults with refractory STC.	60 participants (40 female). 30 received FMT plus conventional therapy, 30 received conventional therapy.	FMT plus conventional therapy resulted in a clinical cure rate of 36.7% vs. 13.3% (*p* = 0.04) compared with conventional therapy alone. Treatment compared with control was also associated with an increased number of CSBMs per week (3.2 vs. 2.1, *p* = 0.001) and colonic transit time (58.5 vs. 73.6 h, *p* < 0.00001).
Dinning et al. [65] 2015 RCT	SNS	Adults with medically refractory STC confirmed by scintigraphy.	Of 59 participants who underwent peripheral nerve evaluation to assess for suitability for permanent SNS, 55 participants (51 females) proceeded with permanent SNS insertion and were included. All patients received both actual and sham stimulations in a cross-over design.	There was no significant difference with either supraseonsory or subsensory stimulation compared with sham stimulations in any of the outcome measures.
Zerbib et al. [67] 2017 RCT	SNS	Adults with medically refractory CIC. All patients underwent assessment of colonic transit times using radio-opaque marker test. 28/36 of the initial participants, and 16/20 of those who progressed to permanent SNS, were classified as STC.	Of 36 participants (34 female) who underwent peripheral nerve evaluation to assess for suitability for permanent SNS, 20 responded and received a permanent SNS and were included. All patients received both actual and sham stimulations in a cross-over design.	There was no significant difference between on- and off- periods of stimulation in any of the outcomes measured.
Yiannakou et al. [68] 2019 RCT	SNS	Adults with medically refractory CIC. All patients underwent assessment of colonic transit times. 30/45 of initial participants were classified as STC.	Of the 45 participants (43 female) who underwent peripheral nerve evaluation to assess for suitability for permanent SNS, 29 were responders, 2/29 did not proceed, and 27 ultimately received a permanent SNS and were included. All patients received both actual and sham stimulations in a cross-over design.	There was no significant difference between on- and off- periods of stimulation in any of the outcomes measured. Additionally, there was no difference between those who were discriminate and indiscriminate responders during the peripheral nerve evaluation.
Ng et al. [69] 2016 Systematic review	TES	Children with STC confirmed by scintigraphy.	10 studies reporting on a single RCT cohort of 42 children (18 girls) aged 8–18 years, with additional data from their subsequent long-term studies. 21 received TES, 21 received sham stimulation.	TES was associated with a significantly reduced colonic transit time compared with sham stimulation (mean difference 1.05, 95%CI 0.36–1.74). There was no statistical difference between TES and sham stimulation in terms of CSBM/week, soiling or QOL.
Yang et al. [70] 2017 RCT	TES	Women with STC.	28 participants (all female). 14 received TES, 14 received sham stimulation.	TES improved symptoms scores and frequency of SBMs compared with sham stimulation (*p* < 0.05).
Martellucci and Valeri. [71] 2013 Pilot study	Colonic pacing	Adults with medically refractory STC.	Two participants (both female). Both underwent intramuscular electrode placement for colonic pacing.	Number of SBM/week improved from 0.3 to 3.5 in one patient, and 0.5 to 2.5 in the other. Both patients were able to subsequently cease all conventional therapy for constipation and there were no complications.
Peng et al. [73] 2013 RCT	Acupuncture		128 participants. 64 received deep puncture, 33 shallow puncture, and 31 western medication.	Defecation frequency improved from 1.8 to 3.9 SBMs/week with deep puncture acupuncture but did not meet statistical significance (*p* > 0.05). Deep puncture acupuncture was significantly associated with improved defecation frequency to 3.5 SBMs/week at the six month follow up visit (*p* < 0.05).
Lees et al. [75] 2004 Cohort	ACE	Medically refractory CIC (combination of STC, DD, mixed STC/DD patients)	32 participants (26 female) Median age 35. All received ACE.	28/32 required further conduit procedure (19/32 reversed). Satisfactory ACE function achieved in 47%. 12 ultimately went on to surgery (colectomy/ileostomy). Further surgical interventions not affected by prior caecostomy.
Rongen et al. [76] 2001 Cohort	ACE	Medically refractory STC	12 participants (8 female) Mean age 43. All received ACE.	Median defecation frequency improved from 1/week to 1/day. 4/12 ultimately required colectomy. Further surgery not compromised by preceding caecostomy.
Knowles et al. [78] 2017 Systematic review	Surgery	Patients undergoing colectomy for medically refractory STC.	40 studies including 2045 participants. All patients received surgery.	Colectomy resulted in a global satisfaction rate of 86% (range 81–89%). Peri-operative complications occurred in 24.4% (range 17.8–31.7%), with a mortality rate of 0.4%. Abdominal pain and bloating present in 20–50%. Persistent constipation present in 10–30%. Diarrhoea and/or incontinence in 5–15%.

## Data Availability

Data sharing not applicable.

## References

[1] Suares N.C., Ford A.C. (2011). Prevalence of, and risk factors for, chronic idiopathic constipation in the community: Systematic review and meta-analysis. Am. J. Gastroenterol..

[2] Andromanakos N.P., Pinis S.I., Kostakis A.I. (2015). Chronic severe constipation: Current pathophysiological aspects, new diagnostic approaches, and therapeutic options. Eur. J. Gastroenterol. Hepatol..

[3] Bharucha A.E., Pemberton J.H., Locke G.R. (2013). American Gastroenterological Association technical review on constipation. Gastroenterology.

[4] Black C.J., Ford A.C. (2018). Chronic idiopathic constipation in adults: Epidemiology, pathophysiology, diagnosis and clinical management. Med. J. Aust..

[5] Peery A.F., Crockett S.D., Barritt A.S., Dellon E.S., Eluri S., Gangarosa L.M., Jensen E.T., Lund J.L., Pasricha S., Runge T. (2015). Burden of Gastrointestinal, Liver, and Pancreatic Diseases in the United States. Gastroenterology.

[6] El-Salhy M. (2003). Chronic idiopathic slow transit constipation: Pathophysiology and management. Colorectal Dis..

[7] Andrews C.N., Storr M. (2011). The pathophysiology of chronic constipation. Can. J. Gastroenterol..

[8] Shahid S., Ramzan Z., Maurer A.H., Parkman H.P., Fisher R.S. (2012). Chronic idiopathic constipation: More than a simple colonic transit disorder. J. Clin. Gastroenterol..

[9] Bassotti G., Roberto G.D., Sediari L., Morelli A. (2004). Toward a definition of colonic inertia. World J. Gastroenterol..

[10] Drossman D.A., Hasler W.L. (2016). Rome IV-Functional GI Disorders: Disorders of Gut-Brain Interaction. Gastroenterology.

[11] Huizinga J.D., Hussain A., Chen J.H. (2021). Interstitial cells of Cajal and human colon motility in health and disease. Am. J. Physiol. Gastrointest. Liver Physiol..

[12] Feldman M., Friedman L.S., Brandt L.J. (2020). Sleisenger and Fordtran’s Gastrointestinal and Liver Disease: Pathophysiology, Diagnosis, Management.

[13] Sharma A., Rao S. (2017). Constipation: Pathophysiology and Current Therapeutic Approaches. Handb. Exp. Pharmacol..

[14] Tornblom H., Lang B., Clover L., Knowles C.H., Vincent A., Lindberg G. (2007). Autoantibodies in patients with gut motility disorders and enteric neuropathy. Scand. J. Gastroenterol..

[15] Rao S.S., Sadeghi P., Beaty J., Kavlock R. (2004). Ambulatory 24-hour colonic manometry in slow-transit constipation. Am. J. Gastroenterol..

[16] Thayalasekeran S., Ali H., Tsai H.H. (2013). Novel therapies for constipation. World J. Gastroenterol..

[17] Bharucha A.E., Wald A. (2019). Chronic Constipation. Mayo Clin. Proc..

[18] Bharucha A.E., Lacy B.E. (2020). Mechanisms, Evaluation, and Management of Chronic Constipation. Gastroenterology.

[19] Wlodarczyk J., Wasniewska A., Fichna J., Dziki A., Dziki L., Wlodarczyk M. (2021). Current Overview on Clinical Management of Chronic Constipation. J. Clin. Med..

[20] Tian H., Chen Q., Yang B., Qin H., Li N. (2021). Analysis of Gut Microbiome and Metabolite Characteristics in Patients with Slow Transit Constipation. Dig. Dis. Sci..

[21] Bharucha A.E., Philips S.F. (2001). Slow-transit Constipation. Curr. Treat Options Gastroenterol..

[22] Roszkowska A., Pawlicka M., Mroczek A., Balabuszek K., Nieradko-Iwanicka B. (2019). Non-Celiac Gluten Sensitivity: A Review. Medicina.

[23] Lewis S.J., Heaton K.W. (1997). Stool form scale as a useful guide to intestinal transit time. Scand. J. Gastroenterol..

[24] Saad R.J., Rao S.S., Koch K.L., Kuo B., Parkman H.P., McCallum R.W., Sitrin M.D., Wilding G.E., Semler J.R., Chey W.D. (2010). Do stool form and frequency correlate with whole-gut and colonic transit? Results from a multicenter study in constipated individuals and healthy controls. Am. J. Gastroenterol..

[25] Tantiphlachiva K., Rao P., Attaluri A., Rao S.S. (2010). Digital rectal examination is a useful tool for identifying patients with dyssynergia. Clin. Gastroenterol. Hepatol..

[26] Southwell B.R., Clarke M.C., Sutcliffe J., Hutson J.M. (2009). Colonic transit studies: Normal values for adults and children with comparison of radiological and scintigraphic methods. Pediatr. Surg. Int..

[27] Li Y.W., Yu Y.J., Fei F., Zheng M.Y., Zhang S.W. (2019). High-resolution colonic manometry and its clinical application in patients with colonic dysmotility: A review. World J. Clin. Cases.

[28] Jensen M.M., Pedersen H.E., Clemmensen K.K.B., Ekblond T.S., Ried-Larsen M., Faerch K., Brock C., Quist J.S. (2023). Associations Between Physical Activity and Gastrointestinal Transit Times in People with Normal Weight, Overweight, and Obesity. J. Nutr..

[29] Song B.K., Cho K.O., Jo Y., Oh J.W., Kim Y.S. (2012). Colon transit time according to physical activity level in adults. J. Neurogastroenterol. Motil..

[30] Oettle G.J. (1991). Effect of moderate exercise on bowel habit. Gut.

[31] Robertson G., Meshkinpour H., Vandenberg K., James N., Cohen A., Wilson A. (1993). Effects of exercise on total and segmental colon transit. J. Clin. Gastroenterol..

[32] Suares N.C., Ford A.C. (2011). Systematic review: The effects of fibre in the management of chronic idiopathic constipation. Aliment. Pharmacol. Ther..

[33] Wald A. (2002). Slow Transit Constipation. Curr. Treat Options Gastroenterol..

[34] Dimidi E., Christodoulides S., Fragkos K.C., Scott S.M., Whelan K. (2014). The effect of probiotics on functional constipation in adults: A systematic review and meta-analysis of randomized controlled trials. Am. J. Clin. Nutr..

[35] Chang L., Chey W.D., Imdad A., Almario C.V., Bharucha A.E., Diem S., Greer K.B., Hanson B., Harris L.A., Ko C. (2023). American Gastroenterological Association-American College of Gastroenterology Clinical Practice Guideline: Pharmacological Management of Chronic Idiopathic Constipation. Gastroenterology.

[36] Lee-Robichaud H., Thomas K., Morgan J., Nelson R.L. (2010). Lactulose versus Polyethylene Glycol for Chronic Constipation. Cochrane Database Syst. Rev..

[37] Morishita D., Tomita T., Mori S., Kimura T., Oshima T., Fukui H., Miwa H. (2021). Senna Versus Magnesium Oxide for the Treatment of Chronic Constipation: A Randomized, Placebo-Controlled Trial. Am. J. Gastroenterol..

[38] Kamm M.A., Mueller-Lissner S., Wald A., Richter E., Swallow R., Gessner U. (2011). Oral bisacodyl is effective and well-tolerated in patients with chronic constipation. Clin. Gastroenterol. Hepatol..

[39] Mueller-Lissner S., Kamm M.A., Wald A., Hinkel U., Koehler U., Richter E., Bubeck J. (2010). Multicenter, 4-week, double-blind, randomized, placebo-controlled trial of sodium picosulfate in patients with chronic constipation. Am. J. Gastroenterol..

[40] Muller-Lissner S.A., Kamm M.A., Scarpignato C., Wald A. (2005). Myths and misconceptions about chronic constipation. Am. J. Gastroenterol..

[41] Wald A. (2003). Is chronic use of stimulant laxatives harmful to the colon?. J. Clin. Gastroenterol..

[42] Acosta A., Camilleri M. (2014). Elobixibat and its potential role in chronic idiopathic constipation. Ther. Adv. Gastroenterol..

[43] Nakajima A., Taniguchi S., Kurosu S., Gillberg P.G., Mattsson J.P., Camilleri M. (2019). Efficacy, long-term safety, and impact on quality of life of elobixibat in more severe constipation: Post hoc analyses of two phase 3 trials in Japan. Neurogastroenterol. Motil..

[44] Camilleri M., Piessevaux H., Yiannakou Y., Tack J., Kerstens R., Quigley E.M.M., Ke M., Da Silva S., Levine A. (2016). Efficacy and Safety of Prucalopride in Chronic Constipation: An Integrated Analysis of Six Randomized, Controlled Clinical Trials. Dig. Dis. Sci..

[45] Huang X., Lv B., Zhang S., Fan Y.H., Meng L.N. (2012). Itopride therapy for functional dyspepsia: A meta-analysis. World J. Gastroenterol..

[46] Ford A.C., Suares N.C. (2011). Effect of laxatives and pharmacological therapies in chronic idiopathic constipation: Systematic review and meta-analysis. Gut.

[47] Emmanuel A.V., Roy A.J., Nicholls T.J., Kamm M.A. (2002). Prucalopride, a systemic enterokinetic, for the treatment of constipation. Aliment. Pharmacol. Ther..

[48] Tack J., Coremans G., Janssens J. (1995). A risk-benefit assessment of cisapride in the treatment of gastrointestinal disorders. Drug Saf..

[49] Ueno N., Inui A., Satoh Y. (2010). The effect of mosapride citrate on constipation in patients with diabetes. Diabetes Res. Clin. Pract..

[50] Liu Z., Sakakibara R., Odaka T., Uchiyama T., Uchiyama T., Yamamoto T., Ito T., Asahina M., Yamaguchi K., Yamaguchi T. (2005). Mosapride citrate, a novel 5-HT4 agonist and partial 5-HT3 antagonist, ameliorates constipation in parkinsonian patients. Mov. Disord..

[51] Morrow A. (2022). ZELNORM^®^ (Tegaserod) Notice of Withdrawal from Market. Alfasigma USA, Inc. https://www.myzelnorm.com/assets/pdfs/Press%20Release%20on%20Notice%20of%20Withdrawal.pdf.

[52] Taghavi S.A., Shabani S., Mehramiri A., Eshraghian A., Kazemi S.M., Moeini M., Hosseini-Asl S.M., Saberifiroozi M., Alizade-Naeeni M., Mostaghni A.A. (2010). Colchicine is effective for short-term treatment of slow transit constipation: A double-blind placebo-controlled clinical trial. Int. J. Colorectal Dis..

[53] Roarty T.P., Weber F., Soykan I., McCallum R.W. (1997). Misoprostol in the treatment of chronic refractory constipation: Results of a long-term open label trial. Aliment. Pharmacol. Ther..

[54] Bassotti G., Chiarioni G., Vantini I., Morelli A., Whitehead W.E. (1998). Effect of different doses of erythromycin on colonic motility in patients with slow transit constipation. Z. Gastroenterol..

[55] Sen A., Chokshi R. (2023). Update on the Diagnosis and Management of Acute Colonic Pseudo-obstruction (ACPO). Curr. Gastroenterol. Rep..

[56] Wilkie B.D., Noori J., Johnston M., Woods R., Keck J.O., Behrenbruch C. (2023). Pyridostigmine in chronic intestinal pseudo-obstruction—A systematic review. ANZ J. Surg..

[57] O’Dea C.J., Brookes J.H., Wattchow D.A. (2010). The efficacy of treatment of patients with severe constipation or recurrent pseudo-obstruction with pyridostigmine. Colorectal Dis..

[58] Law N.M., Bharucha A.E., Undale A.S., Zinsmeister A.R. (2001). Cholinergic stimulation enhances colonic motor activity, transit, and sensation in humans. Am. J. Physiol. Gastrointest. Liver Physiol..

[59] Soufi-Afshar I., Moghadamnia A., Bijani A., Kazemi S., Shokri-Shirvani J. (2016). Comparison of pyridostigmine and bisacodyl in the treatment of refractory chronic constipation. Casp. J. Intern Med..

[60] Bharucha A.E., Low P., Camilleri M., Veil E., Burton D., Kudva Y., Shah P., Gehrking T., Zinsmeister A.R. (2013). A randomised controlled study of the effect of cholinesterase inhibition on colon function in patients with diabetes mellitus and constipation. Gut.

[61] Ahuja N.K., Mische L., Clarke J.O., Wigley F.M., McMahan Z.H. (2018). Pyridostigmine for the treatment of gastrointestinal symptoms in systemic sclerosis. Semin. Arthritis Rheum..

[62] Ly A., Rahman M., Song D. (2022). Pyridostigmine as an Effective Treatment for Atonic Colon in Parkinson’s Disease (P13-11.007). Neurology.

[63] Tian H., Ge X., Nie Y., Yang L., Ding C., McFarland L.V., Zhang X., Chen Q., Gong J., Li N. (2017). Fecal microbiota transplantation in patients with slow-transit constipation: A randomized, clinical trial. PLoS ONE.

[64] Ding C., Fan W., Gu L., Tian H., Ge X., Gong J., Nie Y., Li N. (2018). Outcomes and prognostic factors of fecal microbiota transplantation in patients with slow transit constipation: Results from a prospective study with long-term follow-up. Gastroenterol. Rep..

[65] Dinning P.G., Hunt L., Patton V., Zhang T., Szczesniak M., Gebski V., Jones M., Stewart P., Lubowski D.Z., Cook I.J. (2015). Treatment efficacy of sacral nerve stimulation in slow transit constipation: A two-phase, double-blind randomized controlled crossover study. Am. J. Gastroenterol..

[66] Patton V., Stewart P., Lubowski D.Z., Cook I.J., Dinning P.G. (2016). Sacral Nerve Stimulation Fails to Offer Long-term Benefit in Patients with Slow-Transit Constipation. Dis. Colon Rectum.

[67] Zerbib F., Siproudhis L., Lehur P.A., Germain C., Mion F., Leroi A.M., Coffin B., Le Sidaner A., Vitton V., Bouyssou-Cellier C. (2017). Randomized clinical trial of sacral nerve stimulation for refractory constipation. Br. J. Surg..

[68] Yiannakou Y., Etherson K., Close H., Kasim A., Mercer-Jones M., Plusa S., Maier R., Green S., Cundall J., Knowles C. (2019). A randomized double-blinded sham-controlled cross-over trial of tined-lead sacral nerve stimulation testing for chronic constipation. Eur. J. Gastroenterol. Hepatol..

[69] Ng R.T., Lee W.S., Ang H.L., Teo K.M., Yik Y.I., Lai N.M. (2016). Transcutaneous electrical stimulation (TES) for treatment of constipation in children. Cochrane Database Syst. Rev..

[70] Yang Y., Yim J., Choi W., Lee S. (2017). Improving slow-transit constipation with transcutaneous electrical stimulation in women: A randomized, comparative study. Women Health.

[71] Martellucci J., Valeri A. (2014). Colonic electrical stimulation for the treatment of slow-transit constipation: A preliminary pilot study. Surg. Endosc..

[72] Wang L., Xu M., Zheng Q., Zhang W., Li Y. (2020). The Effectiveness of Acupuncture in Management of Functional Constipation: A Systematic Review and Meta-Analysis. Evid. Based Complement. Alternat. Med..

[73] Peng W.N., Wang L., Liu Z.S., Guo J., Cai H.J., Ni J.N., Duan J.X., Yang D.L. (2013). Analysis on follow-up efficacy and safety of slow transit constipation treated with individualized deep puncture at Tianshu (ST 25): A multi-central randomized controlled trial. Zhongguo Zhen Jiu.

[74] Emmett C.D., Close H.J., Yiannakou Y., Mason J.M. (2015). Trans-anal irrigation therapy to treat adult chronic functional constipation: Systematic review and meta-analysis. BMC Gastroenterol..

[75] Lees N.P., Hodson P., Hill J., Pearson R.C., MacLennan I. (2004). Long-term results of the antegrade continent enema procedure for constipation in adults. Colorectal Dis..

[76] Rongen M.J., van der Hoop A.G., Baeten C.G. (2001). Cecal access for antegrade colon enemas in medically refractory slow-transit constipation: A prospective study. Dis. Colon Rectum.

[77] Chu D.I., Balsara Z.R., Routh J.C., Ross S.S., Wiener J.S. (2013). Experience with glycerin for antegrade continence enema in patients with neurogenic bowel. J. Urol..

[78] Knowles C.H., Grossi U., Horrocks E.J., Pares D., Vollebregt P.F., Chapman M., Brown S., Mercer-Jones M., Williams A.B., Yiannakou Y. (2017). Surgery for constipation: Systematic review and practice recommendations: Graded practice and future research recommendations. Colorectal Dis..

[79] Mollen R.M., Hopman W.P., Oyen W.J., Kuijpers H.H., Edelbroek M.A., Jansen J.B. (2001). Effect of subtotal colectomy on gastric emptying of a solid meal in slow-transit constipation. Dis. Colon Rectum.

